# Editorial

**Published:** 1897-01

**Authors:** 


### Editorial.

THIS number begins the twenty-seventh volume of the RECORD.

It is a satisfaction to us to know that at the end of twenty-six years, the Journal is as strong at every point as it has ever been. The last year was one of storm and stress, but we have come out at the end, with a whole skin and even on terms of cordiality with the printers. The coming year is full of promise for us. Our ambition lies in the direction of a home of of our own, canvassers in the field, and workers at home. Atlanta is fast becoming the medical center of the South. Its schools are up-to-date, well equipped and can give a student as good and as practical an education as can be got elsewhere. The physicians of Atlanta are mostly young men, progressive, energetic, imbued with the same spirit that effected the upbuilding of the city. A generous rivalry spurs them on in their efforts to attain proficiency, and there are already not a few for whose opinions the profession in the United States has the greatest respect, and whose work entitles them to rank among the leaders. There is no reason why Atlanta and the territory of whieh she is the focus cannot support a medical journal with a circulation of ten thousand. We want to bring the SOUTHERN MEDICAL RECORD up to that high estate, and we look to the South and Southwest for support. Patronize the home journal, take a pride in it like you do in your court-house or your church. We profit by it, of course, but not much. There is an infinity of hard work, and but small gain in medical journalism. The subscriber it is who reaps the reward by getting a thoroughly good medical publication for a trifling sum of money and no personal exertion, rather than a meager starveling, depending upon the crumbs thrown to it by advertisers to eke its living out.

Nothing in the world is so helpful as encouragement, or so grateful or so cheering. We are working for the good and advancement of the medical profession in the South, and we ask for its co-operation.

Walker (Pediatrics, Oct. 15, 1896) reports seven cases of diphtheria treated with antitoxine, all terminating in recovery. When the diagnosis is clear, he gives, immediately, 1,000 or 1,500 units of the serum, and orders the throat and nose to be kept clean with a solution of bichloride $\frac{1}{5000}$ or of potassium permanganate $\frac{1}{4000}$. In infants the solution should be injected into the nostrils; a spray is most advantageously used in older children. At the end of twenty-four hours he advises a second injection and regards this as generally sufficient. If the symptoms do not point to convalescence at the end of forty-eight hours, a third and even a fourth injection may become necessary. The anti-streptococcic serum should be resorted to in cases of pseudo-diphtheria, and in cases where the membrane shows no tendency to become detached at the end of twenty-four hours. He prefers the serum prepared by Park, Davis & Co., and approves of their method of dispensing it in bulbs, each bulb representing a dose. The serum is tightly concentrated and, being hermetically sealed, will last indefinitely. As to medicinal treatment, the ordinary remedies needed to combat concomitant symptoms are recommended and a fluid diet, principally milk, is advised.

---

Dr. Augustin H. Goelet, Professor of Gynecology in the New York School of Clinical Medicine (*Clinical Recorder*, July, 1896), believes that the best method of closing the abdominal wound after cœliotomy is to use a continuous suture of fine (No. 1) chromic catgut for uniting the peritoneum, and to include with this suture the muscle, but omit the fascia. Next, deep sustaining interrupted sutures of silkworm gut are inserted. These are made to include the skin fascia and muscular layer. Before tying these the fascia is united separately with a continuous suture of the same fine chromic catgut.

The silkworm gut sutures are now tied, the surface washed off and dried carefully.

The ideal dressing for the wound is one which has no disagreeable odor, and will keep it perfectly dry. This will prevent germ propagation. He now uses a boro-phenate of bismuth, known as markasol, which has given more satisfaction than anything else that has been employed.

This is antiseptic without being irritating, and is slightly absorbent and astringent. It will absorb the first oozing from the wound, but holds in contact with the margin of the wound the protective lymph which is thrown out to favor union. It is dusted plentifully over the wound, covering it and the sutures completely; over this is placed a layer of plain sterilized absorbent gauze, and over this several layers of absorbent cotton, which is held in place by strips of rubber adhesive plaster (nearly encircling the body), and a many-tailed bandage.

This dressing may be left undisturbed until the sutures are removed. Then the same powder is again used and a similar cover dressing reapplied.

---

### ON CONGENITAL ELEPHANTIASIS.

Dr. Moncorvo claims to have been the first to demonstrate the frequency of elephantiasis in youth, contrary to the received opinion up to this date. He later on insisted in proving that the evil can very well be developed even during the course of the fœtal life. He has before published ten cases of this character and relates in the present paper an abbreviated history of two others, in one of which the disease affected the face, a part that is not commonly attacked. In Dr. Moncorvo's opinion this neoplasia arises from an inflammation of the lymphatic vessels, which, according to his own investigations and those of Moncorvo, Jr., generally proceed from the presence of the streptococcus of Fehleisen, which must pass from the maternal system to that of the fœtus through the channel of the placenta.

---

### THE JOURNAL OF NERVOUS AND MENTAL DISEASES.

The management announces the following arrangement of the staff for 1897. Editors: Dr. Chas. L. Dana, Dr. F. X. Dercum, Dr. Philip Coombs Knapp, Dr. Chas. K. Mills, Dr. Jas. J. Putnam, Dr. B. Sachs, Dr. M. Allen Starr. Associate editors: Dr. Philip Meirowitz, Dr. Wm. G. Spiller. Managing editor, Dr. Chas. Henry Brown, 25 West 45th St., New York, to whom address all editorial and business communications.

